# Effects of dietary nutrients on volatile breath metabolites

**DOI:** 10.1017/jns.2013.26

**Published:** 2013-10-31

**Authors:** Olawunmi A. Ajibola, David Smith, Patrik Španěl, Gordon A. A. Ferns

**Affiliations:** 1 Guy Hilton Research Centre, Institute for Science and Technology in Medicine, University of Keele, Thornburrow Drive, Hartshill, Stoke-on-Trent ST4 7QB, UK; 2 J. Heyrovský Institute of Physical Chemistry, Academy of Sciences of the Czech Republic, Dolejskova 3, 182 23, Prague 8, Czech Republic; 3 Division of Medical Education, Brighton and Sussex Medical School, Mayfield House, University of Brighton, Brighton BN1 9PH, UK

**Keywords:** Breath analysis, Selected ion flow tube-MS, Macronutrients, Micronutrients, Gut flora, ppbv, parts per billion by volume, PTR, proton transfer reaction, SIFT, selected ion flow tube, VOC, volatile organic compounds

## Abstract

Breath analysis is becoming increasingly established as a means of assessing metabolic, biochemical and physiological function in health and disease. The methods available for these analyses exploit a variety of complex physicochemical principles, but are becoming more easily utilised in the clinical setting. Whilst some of the factors accounting for the biological variation in breath metabolite concentrations have been clarified, there has been relatively little work on the dietary factors that may influence them. In applying breath analysis to the clinical setting, it will be important to consider how these factors may affect the interpretation of endogenous breath composition. Diet may have complex effects on the generation of breath compounds. These effects may either be due to a direct impact on metabolism, or because they alter the gastrointestinal flora. Bacteria are a major source of compounds in breath, and their generation of H_2_, hydrogen cyanide, aldehydes and alkanes may be an indicator of the health of their host.

## Historical background

The relationship between breath composition and health has been known for many centuries. More than 2500 years ago, the Greek physician, Hippocrates of Cos noted the importance of breath smell in the diagnosis of liver disease, using the term ‘*foetor hepaticus*’ to describe the characteristic breath odour associated with liver failure (Treatise on Breath Odour and Disease, 5th century BC).

The ancient Persian physician and philosopher, Ibn Sina (Avicenna) wrote that ‘...it is the role of the vital force (breath) to maintain a perfect equilibrium within the elements of the body, and between the elements of the body and the environment’ (The Canon of Medicine, 10th century). An important environmental determinant of breath composition is diet. Approximately 40 years ago, Pauling *et al.*^(^^1^^)^ investigated the relationship between breath composition and diet and recognised the potential impact of intestinal flora as a contributing factor to breath composition. Individuals were placed on a defined elemental diet, consisting almost entirely of small molecules that the authors assumed would be absorbed from the upper gastrointestinal tract, and that intestinal flora would be reduced in the lower gastrointestinal tract because of the lack of nutrients reaching them. Using temperature-programmed gas–liquid partition chromatography, the quantitative determination of about 250 substances in a sample of human breath was possible at that time.

## Introduction

Today, using exquisitely sensitive analytical techniques, more than 500 compounds have been reproducibly identified in exhaled breath^(^^2^^)^, though as many as 3000 different compounds have been sporadically detected in breath of different individuals^(^^3^,^4^^)^. It is now possible to measure volatile organic compounds (VOC) in breath with great sensitivity (down to parts per billion by volume; ppbv) and specificity, using MS and related analytical methods. As a consequence, breath analysis now has a number of well-established clinical applications^(^^5^^)^ (Table 1). It also has enormous potential value in metabolic research, particularly when combined with stable isotope labelling. It has, for example, been used in kinetic studies of amino acid metabolism^(^^6^^)^. Breath analysis may also be used for applications that would otherwise be difficult using other techniques, for example in the assessment of whole-body oxidative stress^(^^7^^)^, or cholesterol biosynthesis^(^^8^^)^. Breath analysis may also be a useful adjunct to blood and faecal analysis in the investigation of gut microbiota^(^^9^^)^. The present review briefly outlines the physiological and dietary factors that may have an important impact on breath compounds and the methods used for assessing them, together with the reported concentrations of these compounds in health and disease.

**Table 1.** Established and emerging clinical applications of breath analysis

Breath analysis	References
Breath H_2_ test for carbohydrate metabolism	Rumessen *et al.*^(^^137^^)^; Romagnuolo *et al.*^(^^138^^)^; Eisenmann *et al.*^(^^139^^)^; Bond & Levitt *et al.*^(^^140^,^141^^)^
Breath NO test to monitor therapy for asthma	Eisenmann *et al.*^(^^139^^)^; Taylor *et al.*^(^^142^^)^
Breath CO test for neonatal jaundice	Stevenson *et al.*^(^^143^^)^
Breath test for diagnosis of *Helicobacter pylori*	Romagnuolo *et al.*^(^^138^^)^
Breath test for heart transplant rejection	Phillips *et al.*^(^^144^^)^
Breath NH_3_ has been identified as an indicator of the efficacy of renal dialysis	Endre *et al.*^(^^145^^)^; Rolla *et al.*^(^^146^^)^; Narasimhan *et al.*^(^^147^^)^
Breath H_2_ and the ^13^CO_2_:^12^CO_2_ ratio (following the ingestion of ^13^C-labelled compounds) as related to gastric emptying and bowel transit times	Bond & Levitt *et al.*^(^^141^^)^; Braden *et al.*^(^^148^^)^; Rao *et al.*^(^^149^^)^; Geboes *et al.*^(^^150^^)^
Hydrogen cyanide is released by the pathogen, *Pseudomonas aeruginosa*, and the detection of high concentrations of hydrogen cyanide in breath may be used for the early detection of bacterial infection of children with cystic fibrosis	Shestivska *et al.*^(^^151^^)^; Carroll *et al.*^(^^152^^)^

## Sources of volatile metabolites in exhaled breath

**Fig. 1.**
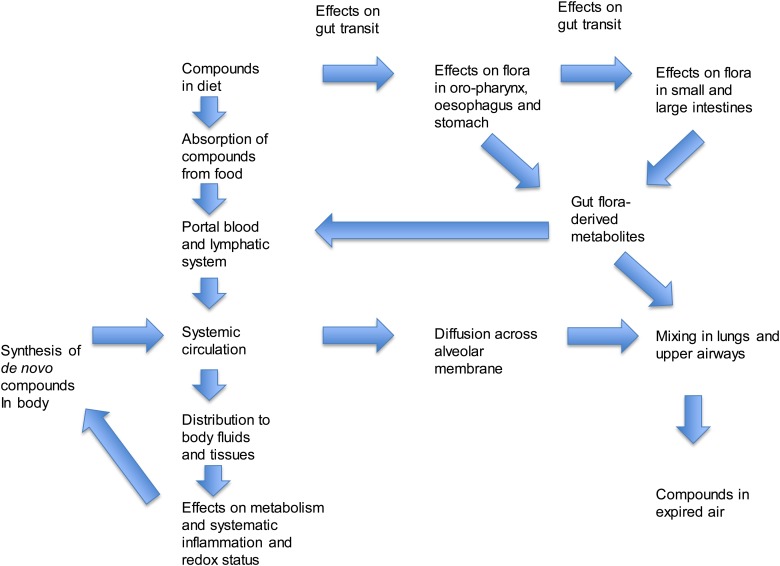
The complex interactions between diet and expired breath metabolites.

Volatile metabolites in exhaled breath are derived from several sources: they may be derived from the environmental inspired air, from cells, including micro-organisms that are located throughout the oral/nasal cavities and the pulmonary system, the upper and lower gastrointestinal tracts and from general human metabolism (Fig. 1)^(^^10–^^12^^)^. For example, NO is present in trace amounts in atmospheric air and is therefore present in the inspired air; it is also an important biological mediator in the vasculature^(^^13^^)^, being derived from the action of NO synthase on the amino acid arginine, and is elaborated at high concentrations by activated inflammatory cells^(^^14^^)^. Its concentrations in expired air can therefore be considerably higher than in inspired air, and may be derived from several sources.

## Physiological variations in breath composition

### Site of exhaled breath sampling

The measured concentrations of several exhaled breath constituents differ significantly depending on their site of breath sampling; whether from the mouth, nose, or the static oral cavity. Some of these compounds, for example NH_3_, ethanol and hydrogen cyanide, are predominantly generated in the oral cavity in healthy subjects^(^^15^,^16^^)^. Hence oral health, including periodontal and dental disease, are potential confounding factors^(^^17^^)^. It has been shown that concentrations of some compounds in the exhaled breath, for example NH_3_ and ethanol, can be increased by sugar and urea mouth washes^(^^18^^)^. Hence, without careful preparation, mouth production of these and other compounds can compromise the quantification of endogenous trace compounds present in the alveolar breath. However, the concentrations of both the urea and sucrose solutions used in these latter studies that proved the enhancement of NH_3_ and ethanol levels were greater than normally present in food and beverages; thus in most situations such severe enhancements will not occur^(^^18^^)^. It is also possible to simultaneously monitor mouth and nasal concentrations of breath compounds to elucidate their source^(^^19^^)^. Furthermore, it is possible to sample end-tidal gas only^(^^20^^)^ by physically filtering out gas from the oral cavity (for example, by using buffered end-tidal sampling), or by data processing *post hoc*^(^^20^,^21^^)^.

### Determinants of inter-individual and intra-individual variation

Intra-individual studies that have been carried out over about 30 d have revealed the temporal variations in the concentrations of several common breath metabolites for several individuals, including: NH_3_, acetone, isoprene, ethanol and acetaldehyde^(^^11^,^22^^)^. Breath NH_3_, acetone and isoprene concentrations were reported to have CV of typically 0·3 over this period. No obvious correlations were found in the distributions of these particular metabolites, except that the NH_3_ levels were greatest in the breath of the oldest subjects^(^^22^^)^. In population (inter-individual) studies over a longer time-frame (6 months), breath methanol levels appeared to have a log-normal distribution for the study population, and did not correlate with age, breath ethanol or ethanol consumed in the previous 24 h; however, there was an inverse correlation with BMI^(^^23^^)^. Breath NH_3_ increased with age, and a weak but significant correlation between breath propanol and acetone levels was reported^(^^24^^)^. Breath isoprene concentrations have been studied in healthy schoolchildren between 7 and 18 years of age, and in this group there was a strong positive association with age^(^^25^^)^, possibly related to growth, or steroid hormone biosynthesis.

### Fasting and the acute effects of feeding on breath content

Effects of the fasted or fed state on breath constituents are complex. Breath acetone, NH_3_, ethanol, isoprene and methanol have been measured during single exhalations whilst fasting and following feeding with a liquid protein-energy meal^(^^26^^)^. Breath acetone concentrations fell from a maximum during fasting, reaching their nadir between 4 and 5 h after feeding. Changes in breath NH_3_ concentrations were biphasic, possibly related to changes in portal blood flow, with a rapid fall to approximately 50 % of their fasting levels before rising to two or three times their baseline values at 5 h^(^^26^^)^. A brief increase in breath ethanol concentrations was found after feeding, and this is probably related to the ethanol content of some foods. Subsequently, breath ethanol levels remained low throughout the experimental period. Isoprene concentrations did not change significantly^(^^26^^)^. Levels of breath ethanol increased if a sweet drink or food had been consumed within 2 h before providing a breath sample, but surprisingly no increase in breath ethanol was apparent when modest alcohol consumption had occurred the previous evening. Endogenous breath ethanol and acetaldehyde levels were not significantly correlated with each other^(^^27^^)^. It has recently been reported that breath hydrogen cyanide may rise following the consumption of food or drink^(^^28^^)^.

### Effect of exercise and the breath cycle on breath content

Alveolar breath isoprene and methyl acetate have been reported to increase immediately after moderate exercise, returning to baseline soon thereafter^(^^29^,^30^^)^. We have recently reported that breath isoprene concentrations rise rapidly after commencing exercise, and then decrease during the period of exercise. Plasma cholesterol levels were not obviously correlated with isoprene concentration in breath. Also, isoprene levels were not found to be directly related to sex, age or BMI in this study of adults^(^^30^^)^. The changes in breath isoprene during exercise have been attributed to changes in tissue fractional perfusion^(^^31^^)^, and the changes in expiratory breath NO observed during exercise have been reported to be due to changes in air flow rate, rather than increased NO production^(^^32^^)^. The exercise-related changes in NH_3_ concentrations in breath exhaled via the nose appear to vary with age, with a several-fold increase in concentrations persisting into the post-exercise period^(^^31^^)^. These changes may be dependent on renal function. Breath composition changes during the breathing cycle. For example, the variation observed in breath acetone appears to be dependent on exhaled volume, but not flow^(^^33^^)^.

### Molecules directly or indirectly derived from food, beverages and medicines

Following the ingestion of some compounds, there are wide inter-individual variations in their appearance in the breath. For example, following the ingestion of eucalyptol, a constituent of proprietary medications, its appearance in breath varies between 1 and 5 h after ingestion, showing wide inter-subject variations too^(^^34^^)^. Green tea was very effective in reducing volatile sulfur compounds (hydrogen sulfide and methyl sulfide) in mouth breath, this being attributed to its disinfectant properties^(^^35^^)^. The kinetics of the acute release of aromas from food or beverages is complex, being dependent on the physiological processes involved in swallowing, the lipid content of the food^(^^36^^)^ and the vapour pressure of the compound^(^^37^^)^. There are a number of volatile compounds in food that may rapidly appear in the breath following their consumption^(^^38^^–^^40^^)^.

### Breath alkanes, smoking, other causes of oxidant stress and dietary antioxidants

Smoking is known to induce a state of oxidative stress that is associated with lipid peroxidation^(^^41^,^42^^)^ and has been shown to be associated with substantial changes in breath composition^(^^43^^)^. Oxidative stress has the potential to damage cells, tissues and organs via the production of reactive oxygen species such as superoxide, H_2_O_2_ and the hydroxyl radical^(^^44^^)^. Oxidative stress may be estimated through breath measurements of biomarkers that include ethane, ethylene and pentane^(^^45–^^52^^)^. Although these hydrocarbons only represent a small and possibly variable proportion of the total amount of peroxidised PUFA, their determination in exhaled breath enables an assessment of oxidative stress *in vivo*^(^^53^^)^. Do *et al.*^(^^54^^)^ have previously reported that non-smokers have very low baseline levels of ethane, whilst ethane production correlated with active (packs per d) and lifelong (pack-years) tobacco consumption. Miller *et al.*^(^^55^^)^ have reported similar findings and also report that breath ethane concentrations are related to the time interval between the last cigarette smoked and breath sampling. Do *et al.*^(^^54^^)^ have also shown that antioxidant vitamin supplementation resulted in attenuation of smoking-related lipid peroxidation with a significant decrease in breath ethane production. Aghdassi & Allard^(^^45^^)^ assessed oxidative stress using breath alkane output and other markers of lipid peroxidation in several conditions associated with inflammation, including smoking. Lipid peroxidation was significantly higher and antioxidant vitamin status significantly lower in smokers compared with non-smokers. β-Carotene or vitamin E supplementation significantly reduced lipid peroxidation, whilst vitamin C supplementation had no significant effect. These findings are consistent with those of Hoshino *et al.*^(^^56^^)^ and Allard *et al.*^(^^57^^)^.

In an animal model of vitamin E deficiency, the increased peroxidation of tissue lipids leads to an increased level of breath pentane^(^^58^^)^. However, in their paper, Gelmont *et al.*^(^^58^^)^ reported that pentane production was also dependent on dietary linoleate. Breath pentane in the study animals was reduced by removal of linoleate from their diet, by starvation, antibiotic treatment or the addition of vitamin C to their food or water. Breath pentane was increased by the removal of vitamin E from the diet. The authors concluded that intestinal bacteria were a major source of breath pentane in addition to endogenous membrane lipid peroxidation^(^^58^^)^. In recent studies we have found, using selected ion flow tube (SIFT)-MS, that breath pentane is elevated in patients with inflammatory bowel disease such as ulcerative colitis^(^^59^^)^.

The effects of a restricted-energy diet have also been investigated in the rat model^(^^60^^)^. A significant decrease in ethane generation was found in the rats receiving an energy-restricted diet compared with those fed *ad libitum*, supporting the hypothesis that energy restriction reduces the level of oxidative stress^(^^61^^)^.

Breath pentane is derived from the oxidation of *n*-3 and *n*-6 fatty acids which appear to be transferred from mother to fetus during pregnancy^(^^62^^)^. However, in women in their last trimester, who have smoked during pregnancy, it has been reported that breath ethane was higher than for a control group of non-smokers, and inversely related to serum vitamin C^(^^63^^)^. Dietary *n*-3 fatty acid supplementation also appeared to increase lipid peroxidation as assessed by breath alkane output, and this was not prevented by co-administration of vitamin E^(^^64^^)^.

## Dietary studies

**Fig. 2.**
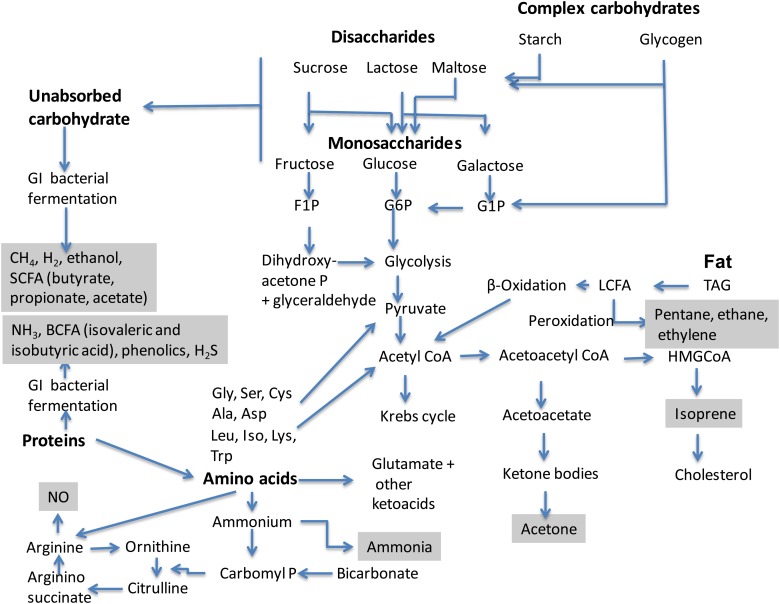
Dietary and metabolic sources of the major metabolites in human breath. GI, gastrointestinal; F1P, fructose 1-phosphate; G6P, glucose 6-phosphate; G1P, glucose 1-phosphate; LCFA, long-chain fatty acids; BCFA, branched-chain fatty acids; HMG, hydroxy methyl glutaryl; carbomyl P, carbomyl phosphate. The grey boxes represent compounds that have been identified in breath.

The effects of dietary constituents on breath composition are complex, as alluded to in Fig. 2. The acute effects of diet on breath have been described briefly above. Medium- and longer-term effects may be mediated by changes of flora in the gastrointestinal tract^(^^65^,^66^^)^ and direct or indirect effects on gastro-caecal transit time^(^^67^,^68^^)^, together with effects on metabolism, systemic inflammation^(^^69–^^71^^)^ and redox state^(^^72^,^73^^)^.

### Effects of macronutrients and dietary energy restriction

#### High- and low-fat diets

Rosenkranz *et al.*^(^^74^^)^ have investigated the acute effects of a high-fat meal on pulmonary function and expiratory NO. They found that a high-fat meal was associated with increased expiratory NO, but had no effect on a systemic marker of inflammation, or pulmonary function in normal individuals, and the authors concluded that a high-fat diet may contribute to inflammation within the airway. Studies in patients with asthma have found that a diet containing a high *n*-6:*n*-3 fatty acid ratio was associated with worsening of asthma control and higher concentrations of NO in exhaled breath^(^^75^^)^.

Ketogenic diets are high in fat, low in carbohydrate and contain adequate levels of protein. Under these conditions fat is metabolised in preference to carbohydrates, and ketone bodies (acetone, acetoacetate and β-hydroxybutyrate) are generated in the liver, leading to ketosis^(^^76^^)^. In these circumstances, expiratory breath acetone concentrations are increased substantially. Even in healthy subjects, breath acetone has been reported to rise more than five-fold following a ketogenic diet^(^^77^,^78^^)^. Breath acetone appears to be indicative of systemic ketosis associated with a ketogenic diet^(^^77^,^79^^)^. Under certain circumstances acetone is reduced to isopropanol by hepatic alcohol dehydrogenase and this then also appears in the breath^(^^80^^)^.

#### Simple carbohydrates and alcohol

H_2_ breath tests have been used for the assessment of carbohydrate malabsorption and abnormal bacterial colonisation of the gut for many years^(^^81^^)^. Basal breath H_2_ is dependent on dietary carbohydrate^(^^82^^)^. H_2_ production in man is primarily dependent upon the delivery of ingested, fermentable substrates to an abundant intestinal flora that is normally present only in the colon. In the normal intestine, more than 99 % of H_2_ production appears to be of colonic origin, but small-bowel production may be increased in a patient with excessive numbers of small-bowel bacteria. H_2_ breath tests are based on the fact that there is no source for H_2_ gas in humans other than bacterial metabolism of carbohydrates. Respiratory H_2_ excretion can therefore be used as an indicator of intestinal H_2_ production. In carbohydrate tolerance tests, different carbohydrates are administered orally and the concentration of H_2_ is measured in expired air. When defective sugar absorption is present, unabsorbed sugars are available in the colon for bacterial fermentation^(^^83^,^84^^)^. Approximately 14 % of the total H_2_ production is excreted by the lungs, and rates of breath H_2_ excretion and production correlates well^(^^85^^)^. Smoking raises and exercise lowers H_2_ concentrations and is therefore not allowed during these tests^(^^85^^)^.

Orocaecal transit time is increased in subjects with alcoholism, but it also appears to be increased among individuals who drink moderate amounts of alcohol as assessed by the H_2_ breath test^(^^86^,^87^^)^. Clearly, this has the potential to alter the occurrence of specific breath constituents and the overall postprandial breath profile. Alcohol is largely metabolised to acetaldehyde by dehydrogenase enzymes, leading to the appearance of high concentrations of acetaldehyde in the breath after alcohol consumption^(^^88^,^89^^)^. Somatic cells and microbes representing normal human gut flora are also able to produce acetaldehyde from ethanol^(^^90^^)^. After the ingestion of alcoholic beverages, there are high local acetaldehyde concentrations in the saliva, gastric juice and the contents of the large intestine. In addition, microbes may produce acetaldehyde endogenously in the absence of exogenous alcohol administration^(^^90^^)^.

#### Complex carbohydrates and fibre

Complex carbohydrate and fibre increase gut transit time and therefore increase the quantity of fermentable, non-absorbed carbohydrate reaching the distal intestine, and hence increase the production of gut-derived H_2_ and CH_4_^(^^91–^^93^^)^. In some groups of subjects, there appears to be an adaptation to high intakes of resistant starch over time and an apparent relationship with insulin sensitivity^(^^94^^)^. Using breath H_2_ analysis, Strocchi & Levitt^(^^95^^)^ found that 5–10 % of starch in wheat, potatoes and maize is not absorbed by healthy subjects, while rice starch is nearly completely absorbed. The physiological effects of dietary fibre are not always predictable from their physicochemical properties^(^^96^^)^. For example, maize fibre has been reported to resist fermentation better than potato fibre and to have a lower digestibility^(^^96^^)^. However, both dietary fibres increased faecal output of DM, neutral sugars and water. Orocaecal transit time is increased by potato fibre, and it is reported to reduce postprandial plasma levels of total and esterified cholesterol. In contrast, maize fibre has been reported to lower fasting blood cholesterol concentrations and increase the non-esterified cholesterol ratio. A class of non-digestible but fermentable oligosaccharides, *trans*-galacto-oligosaccharides, was found to increase the concentration of breath H_2_ and the N density of the faeces^(^^97^^)^, whilst dietary fibre from maize, cassava and amaranth all increased faecal energy loss. Expired breath H_2_ was highest for those individuals consuming maize or cassava^(^^98^^)^. In critically ill patients receiving jejunal feeding with a semi-elemental diet, fibre supplementation appeared to improve microbiota mass and function, being associated with increased carbohydrate fermentation, measured as breath H_2_ and CH_4_^(^^99^^)^.

#### Protein

The ingestion of a high protein-energy meal is associated with some complex changes in breath compounds; the changes in exhaled acetone, NH_3_ and ethanol concentrations^(^^26^^)^ have been discussed above.

#### Effects of entire diets

A diet that is chronically energy restricted is associated with longevity, which is probably related to a reduction in oxidant stress^(^^100^,^101^^)^; such a diet is also associated with low breath ethane concentrations, that again may relate to reduced oxidant stress^(^^102^^)^.

Kundu *et al.* have found that the amount of breath acetone in exhaled breath was correlated with the rate of fat loss^(^^103^^)^ in subjects on a restricted-energy weight-loss programme.

Using a randomised controlled design of the effects of a diet rich in fruit and vegetables, with or without low-fat dairy products for 8 weeks' duration, Miller *et al.*^(^^104^^)^ found that breath ethane was significantly reduced in patients on both fruit- and vegetable-rich diets, but particularly in subjects on a low-fat dairy diet. The endogenous production of methanol is increased after the consumption of fruit^(^^105^^)^, its concentrations increasing by as much as an order of magnitude. This is thought to be due to the degradation of natural pectin (which is esterified with methyl alcohol) in the colon. *In vivo* studies showed that pectin in either a pure form (10 to 15 g) or a natural form (in 1 kg of apples) induces a significant increase of methanol in the breath (and by inference in the blood) of humans^(^^105^^)^ to a level similar to that seen following the consumption of alcohol spirits^(^^106^^)^.

#### Effects of pre- and probiotics

*In vitro* studies of isolated bacterial cultures have demonstrated that the VOC profile that they produce is distinctive, and may be used to differentiate bacterial species^(^^107^^)^. Whilst some of these molecules, for example ethanol and acetone, are produced by their human host, other trace gases, for example H_2_ and indole, are only produced in detectable quantities by bacteria^(^^107^^)^. Furthermore, the gases emitted by bacteria also appear to be dependent on the strain of the bacterial isolate^(^^108^^)^ and culture conditions^(^^109^^)^. Bartram *et al.*^(^^110^^)^ have reported that daily yogurt enriched with *Bifidobacterium longum* and 5 g lactulose/l increased breath H_2_ exhalation and mouth-to-caecum transit time.

SCFA are produced by bacterial fermentation of carbohydrates in the colon, influence gastrointestinal motility^(^^111^^)^, and can affect motility at a distance from their site of production. The mechanisms of action of SCFA on gastrointestinal motility have not been completely elucidated. They may involve systemic humoral and neural pathways as well as local reflexes and myogenic responses.

Cellobiose has a β-1,4 linkage, so it is resistant to hydrolysis by human small-intestinal disaccharidase and, hence, reaches the colon undigested. The excretion of breath H_2_ gas after cellobiose ingestion was found to be significantly greater than after glucose ingestion^(^^112^^)^. In another study, prebiotic treatment increased breath H_2_ excretion by 3-fold and reduced hunger^(^^113^^)^. The AUC for plasma glucagon-like peptide 1 and the volatile release curve for breath-H_2_ excretion measured after the meal were significantly correlated with each other^(^^113^^)^.

#### Dietary micronutrients

Micronutrients have the potential to affect redox status and prevailing inflammation, or they may have direct effects on constituents within breath. Furthermore, lung function (forced expiratory volume) appears to be related to dietary vitamin C^(^^114^^)^ and fruit intake^(^^115^^)^, although the latter study was in children and so the findings may not be the same for adults. Increased concentrations of breath alkanes are associated with reduced antioxidant micronutrient status^(^^45^^)^. Supplementation with a cocktail of antioxidant vitamins (vitamin C, vitamin E and β-carotene) has been reported to be associated with reduced breath pentane in smokers^(^^116^^)^. In contrast, Fe supplements have been found to increase breath ethane concentrations in young women^(^^117^^)^. The amount of breath dimethyl selenide has been reported to increase after the ingestion of Se supplements^(^^118^^)^ and substantial amounts are found in the breath of individuals with Se toxicity^(^^119^^)^, and this may account for the characteristic breath odour in individuals with this condition^(^^119^^)^.

## Principles of measurement

The ability to accurately measure concentrations of trace gases in humid breath has only been possible in the last 20–30 years. GC-MS has been used widely used for breath analysis and continues to be vigorously exploited to great effect for this purpose. In GC-MS, breath samples are collected and volatile compounds extracted and pre-concentrated before offline analysis. Whilst GC-MS has allowed the identification of compounds in breath it is not possible to use this technique in real time^(^^3^,^4^^)^. It is disturbed by the large amount of water vapour present in humid exhaled breath.

More recent analytical advances include SIFT-MS, proton transfer reaction (PTR)-MS and various optical spectroscopic or electronic ‘nose’ devices; these are techniques that have allowed real-time analysis of breath^(^^120–^^123^^)^. Spectroscopic detection methods have been designed to detect specific simple molecules of permanent gases, such as NO and ethane^(^^121^^)^ rather than a profile of VOC in breath, but are amenable to real-time applications. The physicochemical principle of electronic nose devices is that exposure of the detector to specific compounds is associated with a change in surface conductivity of the sensor; however, interpretation may be complicated for humid samples^(^^123^^)^ and they generally lack positive identification.

## Methods commonly used for breath analysis

Those methods used for breath analysis mentioned above are briefly described below and summarised in Table 2. The most widely reported breath analytes are shown in Table 3, together with the methods used to detect them, their concentrations, sources and potential confounding factors.

**Table 2.** Summary of methods used for breath analysis

Method	Principle	References
GC	Sample injected onto a chromatographic column using an inert gaseous mobile phase. The column may be polar or non-polar. The separated compounds are then detected by MS, flame ionisation or ion mobility spectrometry	Phillips^(^^3^^)^, Sanchez & Sachs^(^^153^^)^
PTR-MS and PTR-TOF	VOC are ionised by their reaction with H_3_O^+^. Can be performed without pre-concentration, and can be undertaken online. Compounds identified by mass:charge ratio of characteristic ions, isomers cannot be differentiated by PTR-MS and therefore may require further characterisation. Recent PTR-TOF methods have substantially improved mass resolution and are sensitive down to parts per trillion	Moser *et al.*^(^^125^^)^, Lirk *et al.*^(^^126^^)^, Blake *et al.*^(^^128^^)^, Herbig *et al.*^(^^129^^)^
SIFT-MS	VOC are introduced at a controlled rate and reacted with a precursor ion (H_3_O^+^, NO^+^ or O_2_^+^) in a reaction (flow) tube. The product ions are analysed by quadrupole MS. This technique is sensitive down to parts per billion by volume in real time and online, and has good intra-individual repeatability	Smith & Španěl^(^^120^,^155^,^156^^)^, Wang^(^^131^^)^, Smith *et al.*^(^^11^,^154^^)^, Boshier *et al.*^(^^157^^)^
Chemical sensors (noses)	This method relies on the use of chemical sensors, often arranged as arrays, and linked to a data analysis system for chemical fingerprint analysis with reference to a database	Thaler *et al.*^(^^134^^)^, Oh *et al.*^(^^158^^)^
Chemiluminescence	This can be used if the trace gas reacts with ozone to generate a chemiluminescence signal which can be measured using a photomultiplier tube	Toda & Dasgupta^(^^136^^)^
Optical and laser spectroscopy	Absorbance spectroscopy of the trace gas is assessed using a laser light source in the mid-IR range. Recent improvements in the technique have allowed it to be applied to the real-time measurement of a number of potentially important trace gases including NH_3_, ethane and NO	Wang & Sahay^(^^121^^)^, McCurdy *et al.*^(^^122^^)^, Lewicki *et al.*^(^^159^^)^, Dahnke *et al.*^(^^160^^)^

PTR, proton transfer reaction; TOF, time of flight; VOC, volatile organic compounds; SIFT, selected ion flow tube.

**Table 3.** Summary of breath analytes with reported ranges and sources

Compound	Study	Number of subjects	Method	Concentration (ppbv)	Source	Comments
Acetaldehyde	Diskin *et al.*^(^^22^^)^	5	SIFT-MS	Range 2–5	Ethanol metabolism^(^^90^^)^	Oral microbes Liver
	Fuchs *et al.*^(^^161^^)^	12 lung cancer patients 12 smokers 12 healthy volunteers	GC-MS	Lung cancer: mean >200	Carbohydrate metabolism, ambient air	No significant difference in exhaled acetaldehyde concentrations in all subject groups
	Turner *et al.*^(^^27^^)^	30	SIFT-MS	Mean 24 (range 0–104)	Ethanol metabolism	Increased levels detected with consumption of sweet drinks/food 2 h before
Acetone	Turner *et al.*^(^^24^^)^	30	SIFT-MS H_3_O + O_2_^+^	Mean 477 (range 148–2744)	Decarboxylation of acetoacetate, dehydrogenation of isopropanol	Levels strongly influenced by physiological factors other than diet
	Smith *et al.*^(^^26^^)^	6	SIFT-MS	Pre-meal: range 200–600 5 h Post-meal: mean about 200	Related to blood glucose in some studies^(^^162^^)^	
NH_3_	Diskin *et al.*^(^^22^^)^	5	SIFT-MS H_3_O + O_2_^+^	Range 422–2389	Protein metabolism^(^^163^^)^	Not much day-to-day variation in individuals
	Turner *et al.*^(^^24^^)^	30	SIFT-MS	Mean 833 (range 248–2935)	Protein metabolism	Breath concentrations influenced by age and background air
	Španěl *et al.*^(^^163^^)^	6	SIFT-MS	Range 200–1750		Protein diet
	Smith *et al.*^(^^26^^)^	6	SIFT-MS	Pre-meal: range 300–600 5 h Post-meal: range 600–1800		
Allyl sulfides	Rosen *et al.*^(^^164^^)^	Simulated	Thermal desorption GC-MS		Garlic^(^^165^,^166^^)^	Allicin decomposes in gastric acid leading to the formation of allyl sulfides^(^^167^^)^
Carbon disulfide	Phillips^(^^168^^)^	42	GC-MS	Range 0·005–0·008	Atmospheric^(^^168^^)^	
	Ciaffoni *et al.*^(^^169^^)^		Laser absorption spectroscopy		Gut bacteria^(^^170^^)^	
Carbonyl sulfide	Wysocki *et al.*^(^^171^^)^	Simulated	Pulsed quantum cascade-based sensor		Gut bacteria^(^^170^^)^	
	Halmer *et al.*^(^^172^^)^	Simulated	Mid-cavity leak out spectroscopy			
CO	Middleton & Morice^(^^173^^)^	65	Electronic nose device	Non-smokers: mean 1830 Smokers: mean 17400	Haem catabolism^(^^174^^)^	Formation catalysed by hame oxygenase^(^^175^^)^ and CO production is increased by haemolysis^(^^174^^)^
	Paredi *et al.*^(^^176^^)^	37	Electrochemical	Non-diabetics: mean 2900 Diabetics: range 4–5000		
	Costello *et al.*^(^^177^^)^	10	Electrochemical detector	Non-smokers: mean 2100 (range 600–4900) Smokers: mean 12800 (range 8300–18700)		
Dimethyl sulfide	Tangerman *et al.*^(^^178^^)^		GC	Normal: mean 7·6 (sd 0·6) Cirrhotics: mean 41 (sd 11·5)	Methionine metabolism	Production is dependent on intestinal bacteria^(^^170^^)^
	Azad *et al.*^(^^179^^)^	Simulated	Chemiluminescence			
Ethane	Paredi *et al.*^(^^180^^)^	22	GC	Mean 0·88	Lipid peroxidation^(^^46^,^48^,^50^^)^	The production of ethane and pentane is dependent on antioxidant status^(^^181^,^182^^)^
Ethanol	Diskin *et al.*^(^^22^^)^	5	SIFT-MS H_3_O^+^	Mean 196 (range 0–474)	Gut flora	
	Smith *et al.*^(^^26^^)^	6	SIFT-MS	Range 55–121		
Ethylene	Dumitras *et al.*^(^^183^^)^	1 non-smoker 1 smoker	Photo-acoustic spectroscopy	Mean 20	Lipid peroxidation^(46,48–50)^	
H_2_	Perman *et al.*^(^^184^^)^	221 children 9 adults	GC with electrochemical detection	Mean 7100	Carbohydrate metabolism^(^^81^,^85^^)^	Formed by hydrolytic and saccharolytic bacteria^(^^185^^)^
	Costello *et al.*^(^^177^^)^	10	Electrochemical detector	Mean 9100 (range 300–34000)		Large within-individual variation
Hydrogen cyanide	Schmidt *et al.*^(^^28^^)^	19	Cavity ring down spectroscopy	Range 1·3–6·5	Oxidation of thiocyanate by peroxidase^(^^186^^)^	
	Španěl *et al.*^(^^187^^)^	26	SIFT-MS	Mean 8		
	Enderby *et al.*^(^^188^^)^	16 children with cystic fibrosis 21 children with asthma	SIFT-MS	Mean 13·5 Mean 2·0	Derived from *Pseudomonas aeruginosa*^(^^188^^)^	
Hydrogen sulfide	Costello *et al.*^(^^177^^)^	10	Electrochemical detector	Mean 330 (range 0–1300)	Oral flora	
Isoprene	Turner *et al.*^(^^23^,^30^^)^	20	SIFT-MS NO^+^	Mean 118 (range 0–474)	Cholesterol metabolism^(^^189^,^190^^)^	Exercise^(^^190^^)^ Age Haemodialysis Statin therapy^(^^191^^)^
	Jones *et al.*^(^^192^^)^	16	Thermal desorption GC and diode array UV detection	Range 36–231	Cholesterol metabolism^(^^189^,^190^^)^	No statistical difference in isoprene concentrations between men and women
	Smith *et al.*^(^^25^^)^	200	SIFT-MS	Range 28–54		Age dependent
	Davies *et al.*^(^^193^^)^	19	SIFT-MS	Mean 89 (sd 36)		
	Taucher *et al.*^(^^194^^)^		PTR-MS	Range 100–1000		Children <6 years had values less than adults
Methane	Dryahina *et al.*^(^^195^^)^	75	SIFT-MS O_2_^+^	Range 6–30	Carbohydrate metabolism^(196–198)^	Most produced by *Methanobrevibacter smithii* which uses H_2_ to reduce CO_2_^(^^199^^)^
Methanol	Turner *et al.*^(^^23^^)^	30	SIFT-MS H_3_O^+^	Median 461 (range 32–1684)	Fruit metabolism^(^^105^^)^	Inversely related to BMI Fruit and alcohol
	Taucher *et al.*^(^^106^^)^		PTR-MS	Mean 400		
Methylamine	Marinov *et al.*^(^^200^^)^	Simulated	Near-IR laser spectrometer based on the cavity ring down detection		Protein metabolism	
Methyl nitrate	Novak *et al.*^(^^201^^)^	10 T1DM children	GC offline using electron capture^(^^201^^)^	Range 0·005–0·010	Oxidative processes	Correlation with blood glucose
Methyl mercaptan	Chen *et al.*^(^^170^^)^				Methionine metabolism^(^^170^^)^	
NO	Paredi *et al.*^(^^202^,^203^^)^		Chemiluminescence, following its reaction with O_3_^(^^204^^)^	Mean 6·7	NO synthase and arginine^(205–208)^	Concentration is dependent on exhalation flow rate^(^^209^^)^
	Cobos Barroso *et al.*^(^^210^^)^		Chemiluminescence	Mean 9·7		Concentration in children >17 years predictive of asthma
Pentane	Olopade *et al.*^(^^211^^)^	12 patients (acute asthma) 11 patients (stable asthma) 17 volunteers	GC	Mean 188 (sem 65) Mean 81 (sem 9) Mean 58 (sem 4)	Lipid peroxidation^(^^46^,^48^,^50^^)^	Ethane and pentane production is dependent on antioxidant status^(^^181^,^182^^)^
Propanol		30	SIFT-MS H_3_O^+^	Mean 18 (range 0–135)	Acetone metabolism^(^^212^^)^	Acetone
		46	PTR-MS	Mean 120 (range 50–250)		Natural levels in the body similar to methanol levels

ppbv, Parts per billion by volume; SIFT, selected ion flow tube; PTR, proton transfer reaction; T1DM, type 1 diabetes mellitus.

### Ion mobility spectrometry

The aim of ion mobility spectrometry is to identify trace gases by the mobility of their characteristic gas-phase ions or their derivatives in a buffer/carrier gas^(^^124^^)^. These ions are produced by exposing the carrier gas/trace gas to a radioactive source or electrical discharge when chemical ionisation reactions result in the analytical drifting ions. The movement of these ions is dependent on their mass and molecular geometry, and their dwell times are used to characterise the original mixture of trace gases. Whilst this approach is not recommended for the identification of unknown compounds, it has been used to determine differences in breath metabolite profiles associated with specific diseases^(^^123^^)^.

### Proton transfer reaction-MS and proton transfer reaction-time of flight

In these techniques, precursor hydronium ions (H_3_O^+^) are injected into the buffer gas, which is usually the gas sample to be analysed, and react with the trace gas present in the sample. The precursor ions react with the trace gas species, producing characteristic ion products that are detected and quantified using a down-stream analytical MS. PTR-MS is sensitive down to and below ppbv^(^^125–^^127^^)^. The precursor molecules react with most trace gas molecules to produce a protonated molecule (MH^+^). However, the latter nascent ion may be unstable for some compounds, for example, when M is an alcohol. Furthermore, when the carrier gas in PTR-MS is humid breath, this leads to the formation of cluster ions, for example H_3_O^+^·(H_2_O)_1,2,3_ that may make quantitative analysis more complex, although this cluster ion formation is inhibited by the presence of the axial electric field along the flow tube^(^^128^^)^. In PTR-time-of-flight analysis, ions are accelerated to uniform energy by an electric field, and subsequently traverse a defined distance. The time of flight of the ion is directly related to the ion's mass:charge ratio, and this allows a mass resolution that is substantially better than for conventional PTR-MS^(^^129^^)^. Whilst the original instruments relied on long integration times to attain sufficient sensitivity, recently developed PTR-time of flight instrumentation has improved sensitivity^(^^130^^)^, with integration times of 1 s and a corresponding limit of detection approaching 100 parts per trillion for most compounds, allowing online breath analysis^(^^129^^)^.

### Selected ion flow tube-MS

SIFT-MS combines the fast flow tube technique, chemical ionisation using selected precursor ions, either H_3_O^+^, NO^+^ or O_2_^+^, and quantitative MS that allows online, real-time quantitative analysis of the trace gases (such as ethanol, acetaldehyde, NH_3_, acetone and isoprene, etc.) in single breath exhalations down to concentrations in the ppbv range in a timescale of seconds^(^^131^^)^. SIFT-MS relies on chemical ionisation by the chosen precursor ions of the trace gas molecules in air/breath samples introduced into He carrier gas. These reactions proceed for an accurately defined time, the precursor and product ions being detected and counted by a downstream quadrupole mass spectrometer, thus effecting quantification. Because the absolute concentrations of trace gases in single breath exhalation can be determined by SIFT-MS down to ppbv concentrations, this obviates the need for offline sample collection for the most common breath trace gases. A numerical algorithm allows the calculation, in real time, of absolute concentrations of trace gases, including VOC and water vapour^(^^132^^)^.

### Optical and laser spectroscopic detection

Laser spectroscopic detection techniques have high sensitivity and high selectivity, but also have the advantageous features of near real-time response and low instrument cost. Of approximately thirty-five biomarkers quantified using this method, fourteen species have been analysed in exhaled human breath by high-sensitivity laser spectroscopic techniques, for example acetone, NH_3_, CO_2_, ethane, CH_4_ and NO. The spectral fingerprints of these potentially useful biomarkers span from the UV to the mid-IR spectral regions and the detection limits achieved by the laser techniques range from parts per million by volume to ppbv. Sensors using the laser spectroscopic techniques are already commercially available for a few breath biomarkers, for example CO_2_ and NO^(^^121^^)^.

### Electronic nose detection

Electronic noses, or artificial sensors of volatiles including odorants, have been developed over the last 10 years to perform a variety of identification tasks in various industries. Electronic noses produce a chemical fingerprint of the sample, and this is matched to a reference database^(^^133^^)^. This powerful technology is only beginning to be introduced in the field of medicine, but is promising in its potential to assist in diagnosis^(^^134^^)^.

### Chemiluminescence

Chemiluminescence (CL) is a powerful analytical tool in trace gas analysis. CL monitoring has been used as universal nitrogen and sulfur detectors for GC and capillary electrophoresis^(^^135^^)^. CL detection can be used as the basis of compact and sensitive analysers for real-sample analysis. Isoprene and sulfur compounds in expired breath and atmospheric samples have been successfully measured by coupling to a small collection system. Short-term adsorbent collection enhances the sensitivity and considerably reduces interference. The organosulfur compounds methyl mercaptan and dimethyl sulfide can be separated on the same column that is used for collection^(^^136^^)^.

## Conclusions

Breath analysis is becoming more accessible for clinical and physiological applications. Expired breath is a complex mixture of low-molecular-weight volatile compounds that are derived from diet and endogenous metabolism, or from micro-organisms in the gastrointestinal and respiratory tracts. Metabolic, inflammatory and neoplastic conditions are reported to be associated with characteristic breath profiles, and breath analysis has been promoted as a potentially simple, non-invasive method for screening and monitoring conditions such as asthma, diabetes mellitus and lung cancer. However, there are a number of factors that affect the concentrations of compounds in breath, including diet, physical activity and smoking habit, and it will be important to better understand how these factors influence breath composition as the applications of breath analysis broaden in scope. In order to apply breath analysis to investigations of human nutrition, it would be important to consider any concomitant co-morbidity, including renal and liver dysfunction, neoplastic disease, infection and inflammation. Breath sampling should probably take place under standardised conditions, for example after an overnight fast, and involve diurnal and longitudinal monitoring. A method should be used that is less sensitive to the local release of compounds from the oral cavity. Whichever methods are used should probably also have defined age-related reference ranges.
